# Integration of the Deacetylase SIRT1 in the Response to Nucleolar Stress: Metabolic Implications for Neurodegenerative Diseases

**DOI:** 10.3389/fnmol.2019.00106

**Published:** 2019-04-26

**Authors:** Grzegorz Kreiner, Aynur Sönmez, Birgit Liss, Rosanna Parlato

**Affiliations:** ^1^Department of Brain Biochemistry, Institute of Pharmacology, Polish Academy of Sciences, Kraków, Poland; ^2^Institute of Applied Physiology, University of Ulm, Ulm, Germany; ^3^New College, Oxford University, Oxford, United Kingdom; ^4^Department of Medical Cell Biology, Institute of Anatomy and Cell Biology, University of Heidelberg, Heidelberg, Germany

**Keywords:** sirtuin, oxidative stress, nucleolus, rRNA, p53, neuronal homeostasis, neurodegeneration

## Abstract

Understanding underlying mechanisms of neurodegenerative diseases is fundamental to develop effective therapeutic intervention. Yet they remain largely elusive, but metabolic, and transcriptional dysregulation are common events. Sirtuin 1 (SIRT1) is a nicotinamide adenine dinucleotide (NAD^+^)-dependent lysine deacetylase, regulating transcription, and critical for the cellular adaptations to metabolic stress. SIRT1 regulates the transcription of ribosomal RNA (rRNA), connecting the energetic state with cell growth and function. The activity of the transcription initiation factor-IA (TIF-IA) is important for the transcriptional regulation of ribosomal DNA (rDNA) genes in the nucleolus, and is also sensitive to changes in the cellular energetic state. Moreover, TIF-IA is responsive to nutrient-deprivation, neurotrophic stimulation, and oxidative stress. Hence, both SIRT1 and TIF-IA connect changes in cellular stress with transcriptional regulation and metabolic adaptation. Moreover, they finely tune the activity of the transcription factor p53, maintain mitochondrial function, and oxidative stress responses. Here we reviewed and discussed evidence that SIRT1 and TIF-IA are regulated by shared pathways and their activities preserve neuronal homeostasis in response to metabolic stressors. We provide evidence that loss of rDNA transcription due to altered TIF-IA function alters SIRT1 expression and propose a model of interdependent feedback mechanisms. An imbalance of this signaling might be a critical common event in neurodegenerative diseases. In conclusion, we provide a novel perspective for the prediction of the therapeutic benefits of the modulation of SIRT1- and nucleolar-dependent pathways in metabolic and neurodegenerative diseases.

## Introduction

Sirtuin 1 (SIRT1) is a NAD^+^-dependent deacetylase responsive to metabolic fluctuations and involved in the regulation of a myriad of cellular processes including mitochondrial biogenesis, genomic stability, cellular senescence, and apoptosis (Palacios et al., 2010; Yuan et al., 2016). SIRT1 is considered a key target for treating age-related neurodegenerative diseases, although a deep understanding of its function is required to pin down the beneficial action of its manipulation (Jesko et al., 2017;Fujita and Yamashita, 2018; Silberman, 2018).

Sirtuin 1 has been identified about 20 years ago as the closest human homolog of yeast Sir2 that functions in transcriptional silencing through histone deacetylation, thus setting up a repressive chromatin structure (Sinclair et al., 1998). In yeast Sir2 regulates replicative and organismal aging via epigenetic complexes that can repress rDNA locus recombination maintaining rDNA stability, and increasing longevity (Sinclair and Guarente, 1997; Sinclair et al., 1997). In humans, SIRT1 can de-acetylate the tumor suppressor p53, a key regulator of the response to DNA damage and other stresses leading to genome stability, cell cycle arrest, and cell death (van Leeuwen and Lain, 2009; Lanni et al., 2012).

Interestingly, SIRT1 can also regulate rDNA gene transcription in response to metabolic changes (Grummt and Ladurner, 2008; Murayama et al., 2008). In addition, variation of rDNA copy number can modulate Sir2 expression in budding yeast, further suggesting its conserved function across eukaryotes (Michel et al., 2005).

In general, downregulation of rDNA transcription in response to cellular stresses results in the disruption of the nucleolus, the nuclear compartment hosting rRNA synthesis and pre-ribosome assembly, and site of several hundreds proteins shuttled between nucleolus, nucleus, and cytoplasm (Sharifi and Bierhoff, 2018). This disintegration – a condition known as nucleolar stress and an emerging mechanism associated with cellular stress and age-related neurodegenerative diseases such AD, PD, and HD – may result in the increased stability of p53 (Hetman and Pietrzak, 2012; Parlato and Kreiner, 2013; Chan, 2014;Parlato and Liss, 2014; Parlato and Bierhoff, 2015).

Here we bring together current evidence that SIRT1- and nucleolar-dependent signaling are responsive to changes in the cellular energetic state and activate p53. In connection with this, we discuss their integration in feedback loops, and their dysregulation in neurodegenerative diseases. Based on these premises, we hypothesize that perturbation of rDNA transcription in turn affects SIRT1 function. Based on previous findings and original results, we propose a model of the circuitry that interconnects SIRT1 and rRNA synthesis with each other, with p53 function, and with signaling pathways regulating metabolic adaptation and neuronal survival. Finally, we outline the implications of this model for neurodegenerative pathomechanisms and therapeutic development.

## Sirt1-Dependent Networks in Neurodegenerative Diseases

As indicated above, SIRT1 regulates p53 acetylation states: p53 deacetylation blocks in part its activity, preventing neuronal death (Yuan et al., 2016; Manna et al., 2018). Genetic ablation of SIRT1 in animal models showed that SIRT1 mediates neuronal development, survival, and function (Guarente, 2011; Imai and Guarente, 2014).

Notably, SIRT1 is downregulated in PD, PD with dementia, dementia with Lewy bodies and in AD (Singh et al., 2017). In particular, SIRT1 appears neuroprotective in PD by reducing the formation of alpha-synuclein aggregates upon oxidative stress (Singh et al., 2017). Despite the beneficial effects of compounds activating SIRT1 in PD models, model-dependent differences, and SIRT1 overexpressing transgenic mice also suggest that compounds activating SIRT1 might have other protective functions (Tang, 2017; Fujita and Yamashita, 2018). More recently a putative anti-oxidant and anti-inflammatory neuroprotective mechanism has been linked to SIRT1 activation in AD (Gomes et al., 2018). Moreover, SIRT1 overexpression resulted in neuroprotection against amyloid and tau pathologies and improvement of cognitive functions (Corpas et al., 2017). In agreement with these findings, pharmacological activation of SIRT1 and its overexpression are beneficial in some models of neurodegeneration (Romeo-Guitart et al., 2018). Moreover, the SIRT1 activator resveratrol improved the transcription of genes sustaining mitochondria function in HD models (Naia et al., 2017; Neo and Tang, 2018).

Meanwhile, targeting SIRT1 function for therapeutic purposes presents many challenges (La Spada, 2012; Donmez and Outeiro, 2013; Tang, 2017; Manna et al., 2018). In particular it appears that depending on the cellular context SIRT1 restoration or its inhibition might be beneficial, demanding further research to better understand SIRT1 regulators, and downstream targets. Several positive feedback mechanisms were reported between SIRT1 and its substrates, for example FoxO, a transcription factor that in turn regulates SIRT1 transcription (Kobayashi et al., 2005). Moreover AMPK, whose activity increases upon energy deficits switching off energy-consuming processes, can activate SIRT1 through regulation of the NAD^+^/NADH ratio; in turn SIRT1 activates AMPK (Wang et al., 2011; Price et al., 2012).

Interestingly, in a condition of high glucose SIRT1 activity is low, keeping the balance between an active, and silenced state of the rDNA promoters (Murayama et al., 2008). In low glucose NAD^+^ activates SIRT1 shifting the balance toward a silenced rDNA promoter and inhibition of transcription initiation (Grummt and Ladurner, 2008; Murayama et al., 2008). Another SIRT1 target, PGC-1α is deacetylated in response to resveratrol treatment and promotes rDNA transcription interacting with UBTF1, a member of the RNA Pol I transcriptional machinery (Jesse et al., 2017). The association of PGC-1α with the unmethylated rDNA promoter is prevented by nicotinamide that antagonizes resveratrol, suggesting that PGC-1α de-acetylation is required for its activation and positive effects on rDNA transcription (Jesse et al., 2017). Although the physiological meaning of resveratrol-induced rDNA transcription by de-acetylated PGC-1α remains unclear, reduced RNA Pol I transcription is detected in brain and muscle of PGC-1α knock-out mice, but not in fibroblasts suggesting the tissue-specificity of PGC-1α function on rRNA synthesis (Jesse et al., 2017).

## The Responsiveness of the Nucleolus to the Energetic Cellular State

The nucleolus is a non-membrane-bound nuclear organelle considered an important sensor and mediator of cellular stress responses (Nemeth and Grummt, 2018). rDNA transcription depends on energy availability but also on extracellular factors such as serum starvation, glucose depletion, growth factor and neurotrophin deprivation, oxidative and endoplasmic reticulum stress, and heat shock (Sharifi and Bierhoff, 2018).

A major role in the transcriptional regulation of rDNA genes is played by the transcription factor TIF-IA, that recruits the RNA Pol I to the rDNA promoters, and that is differentially phosphorylated by various kinases including ERK (Zhao et al., 2003), mTOR (Mayer et al., 2004), AMPK (Hoppe et al., 2009), and JNK2 (Mayer et al., 2005;Yuan et al., 2005; Grummt and Langst, 2013).

AMP-activated protein kinase activation adapts rRNA synthesis to nutrient availability and cellular energy status. Indeed low energy level by AMPK-mediated phosphorylation inactivates TIF-IA, but also UBTF1, and in combination with changes in histone acetylation and methylation states decreases rDNA transcription (Grummt and Langst, 2013). Moreover in Drosophila TIF-IA acts as a downstream growth-regulatory target of the TOR pathway and it co-regulates the levels of ribosome components, indicating a master role in the control of protein synthesis and cell metabolism (Grewal et al., 2007).

Experiments performed in murine embryonic fibroblasts conditionally lacking TIF-IA upon Cre recombinase expression revealed that loss of TIF-IA results in nucleolar disruption and p53-dependent apoptosis, and growth arrest (Yuan et al., 2005). Based on these findings, we reasoned that the conditional genetic ablation of TIF-IA in specific cells in mice might represent a unique approach to mimic a condition of nucleolar stress enabling the investigation of context-dependent cellular and molecular consequences of nucleolar stress. By the loss of TIF-IA and induction of nucleolar stress in different neuronal types (e.g., dopaminergic and dopaminoceptive neurons), we showed that nucleolar stress has context-specific neuroprotective/neurotoxic effects resulting in specific progressive neurodegeneration (Parlato et al., 2008; Domanskyi et al., 2011; Rieker et al., 2011; Kiryk et al., 2013; Kreiner et al., 2013; Evsyukov et al., 2017). In particular substantia nigra dopaminergic neurons are more affected than dopaminergic neurons in the ventral tegmental area, recapitulating a typical hallmark of PD (Rieker et al., 2011; Parlato and Liss, 2014).

Although in both dopaminergic and dopaminoceptive neurons mTOR activity is downregulated (Rieker et al., 2011; Kreiner et al., 2013), in dopaminergic neurons mTOR downregulation is neuroprotective (Domanskyi et al., 2011), while in dopaminoceptive neurons restoring mTOR anticipated neuronal loss (Kreiner et al., 2013). Notably, TIF-IA activity is regulated by mTOR and its genetic ablation results in downregulation of mTOR (Rieker et al., 2011; Kreiner et al., 2013). Moreover this negative feedback is context-specific because mTOR is not downregulated in hippocampal neurons lacking TIF-IA (Kiryk et al., 2013).

Nucleolar stress leads to increased p53 stability because ribosomal proteins released from the nucleolus interfere with its turnover by interacting with Mdm2, an E3 ubiquitin ligase responsible for p53 ubiquitination and degradation (Yuan et al., 2005). In striatal neurons p53 increase is associated with its progressive activation via acetylation (Kreiner et al., 2013). However, while ablation of p53 is beneficial for survival of dopaminergic neurons (Rieker et al., 2011), in dopaminoceptive neurons loss of p53 accelerates neurodegeneration, suggesting its transiently neuroprotective functions upon nucleolar stress (Kreiner et al., 2013).

Importantly, we showed that nucleolar stress triggers the loss of mitochondrial activity and increased oxidative stress at a stage preceding neuronal cell death (Rieker et al., 2011; Kreiner et al., 2013). In ventral midbrain dopaminergic neurons loss of TIF-IA leads to downregulation of factors such as yin-yang 1 (YY1) that increases mitochondrial gene transcription in response to mTOR and of one of its targets, the uncoupling protein 2 (UCP2). Cytochrome oxidase activity also decreases before the onset of increased oxidative stress markers such as neuroketals, a marker for reactive oxygen species-induced lipid damage, nitrosylated proteins and 8-hydroxydeoxyguanosine, markers for ROS-induced protein, and DNA damage, respectively (Rieker et al., 2011). Interestingly, dopaminoceptive striatal neurons lacking TIF-IA also show increased oxidative stress (Kreiner et al., 2013).

Importantly, in genetic mouse models based on mutations causing PD, we observed a phasic increase of nucleolar function at early stages. In particular in association with the genetic ablation of PTEN-induced kinase 1 (PINK1/PARK6) and DJ-1 (PARK7) we observed an increased number of nucleoli in dopaminergic neurons (Evsyukov et al., 2017). Notably these pre-symptomatic PD models show compensatory mechanisms sustaining mitochondrial function (Pham et al., 2010; Glasl et al., 2012). The tight association between nucleolar and mitochondrial function is further supported by decreased synthesis of rRNA in neurotoxin-based PD rodent models causing impaired mitochondrial function, showing a cross-talk between nucleoli and mitochondria for the maintenance of their functions (Rieker et al., 2011; Healy-Stoffel et al., 2013).

## Genetic Induction of Nucleolar Stress in Mutant Mice Results in a Reduced Expression of Sirt1

The question whether not only SIRT1 regulates rDNA transcription but also impaired rRNA synthesis alters SIRT1 expression and activity is important to understand the impact of restoring SIRT1, but also nucleolar function, in neurodegenerative disorders.

To this end we took advantage of gene expression profiling data by GeneChip Mouse Genome 430A 2.0 array (Affymetrix, Santa Clara, CA, United States), obtained from conditional knock-out mice lacking TIF-IA in HD-relevant dopaminoceptive striatal neurons, indicated as TIF-IA^D1RCre^ (Kreiner et al., 2013). This mutation results in the loss of rDNA transcription in neurons expressing the D1R affected in HD. Loss of nucleolar integrity was monitored by the distribution of nucleolar protein NPM1 in the nucleoplasm in ca. 80% of the striatal cells (Kreiner et al., 2013), according to the reported expression of the Cre recombinase in the D1RCre transgenic mice (Lemberger et al., 2007).

We compared control and TIF-IA^D1RCre^ mutant mice at 9 and 13 weeks corresponding to a stage before and during neuronal death. We found high level of similarity between genes differentially expressed upon disruption of nucleolar function and genes differentially expressed in HD patients (Kreiner et al., 2013). In fact, nucleolar stress has been reported in various models of HD leading to the hypothesis the mutant Huntingtin affects nucleolar activity e.g., by the interaction with nucleolar NCL (Lee et al., 2011, 2014;Kreiner et al., 2013; Tsoi and Chan, 2013).

**FIGURE 1:**
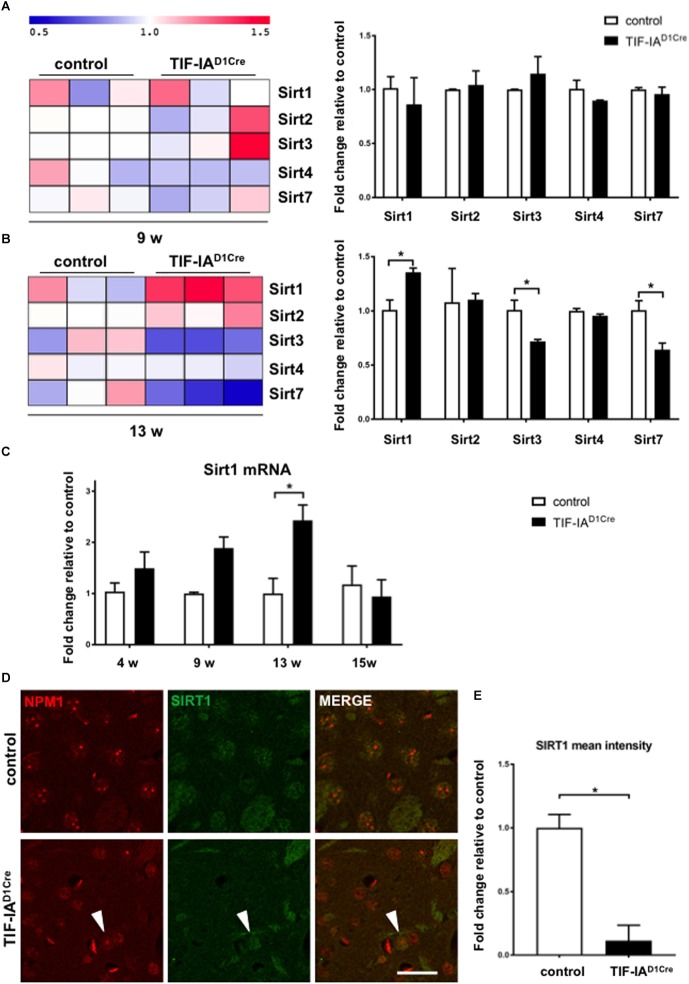
Nucleolar stress affects SIRT1 mRNA and protein expression in the striatum. **(A, B)** Gene expression profiling analysis showing changes of mRNA expression for the sirtuins present on the microarray chip at two different ages (9 and 13 weeks) in control and mutant mice (TIF-IA^D1RCre^) characterized by the genetic conditional ablation of TIF-IA in dopaminoceptive neurons. Heat map visualization of the expression profiling analysis of gene sets encoding for sirtuins reveals upregulation of *Sirt1* mRNA in TIFIA^D1RCre^ mice at 13 weeks and downregulation of *Sirt3* and *Sirt7* at the same time point. Each column represents a single mouse. log2-transformed transcript expression was additionally visualized by a graphical summary of fold change relative to the control mice (^∗^*p* < 0.05 by unpaired two-tailed *t*-test). Briefly, total RNA was dissected from striata of control and mutant mice, checked for its integrity (Bioanalyzer 2100, Agilent, United States), assessed for quantity, and reverse transcribed into cDNA used for microarray hybridization (GeneChip Mouse Genome 430A 2.0 array; Affymetrix, United States). Raw array data were normalized, transformed into expression values, and statistically analyzed using a R/Bioconductor software including Benjamini/Hochberg method to assess false discovery rate. Visualization of heat map was performed with MultiExperiment Viewer (MeV version 4.8.1) to show the expression pattern of genes encoding for sirtuins. The data are stored in the GEO database (http://www.ncbi.nlm.nih.gov/geo/, record number GSE29647). **(C)** Gene expression analysis by qRT-PCR of *Sirt1* mRNA in control and TIF-IA^D1Cre^ mice at different ages using the Chromo4 Platform (Bio-Rad, Hercules, CA, United States). Hypoxanthine-phophoribosyltransferase (Hprt) was chosen as a housekeeping gene (Kreiner et al., 2013). TaqMan inventoried gene expression assays were used: Sirt1 (Mm01168521_m1), Hprt (Mm00446968_m1) (Applied Biosystems/Life Technologies, Carlsbad, CA, United States). Expression changes were calculated as a fold change vs. mean of control samples. Significantly increased levels of *Sirt1* mRNA in the TIF-IA^D1Cre^ mice at 13 weeks (*p* = 0.011 by two-way ANOVA and *post hoc* Sidak’s multiple comparison test); N (control, TIF-IA^D1Cre^) at 4 weeks: 4, 4; at 9 weeks: 3, 4; at 13 weeks: 3, 3; at 15 weeks: 4, 3. Error bars represent SEM. **(D)** Representative images of the immunofluorescence staining for the nucleolar protein NPM1 (red, Millipore, MAB4500) and SIRT1 (green, Santa Cruz, SC-15404) in striatal sections of control and TIF-IA^D1Cre^ mice at 6 weeks. Scale bar: 20 μm. **(E)** Semi-quantitative analysis of the intranuclear SIRT1 signal mean intensity by ImageJ (https://imagej.net/ImageJ) in the nucleus of the TIF-IA^D1Cre^ compared to control mice (*p* = 0.001 by unpaired two-tailed *t*-test after Shapiro-Wilk test for normality). *N* = 4, controls and *N* = 4, TIF-IA^D1Cre^ mice. Error bars represent SEM.

Here we analyzed these gene expression data to detect changes in the expression of various members of the sirtuin family (Figure 1A,B). This analysis revealed upregulation of *Sirt1* mRNA accompanied by downregulation of *Sirt3* and *Sirt7* in 13 week-old mutant mice (Figure 1B). Intriguingly, at 9 weeks, p53 protein levels are not yet significantly increased and the p53 acetylated form increased at 13 weeks (Kreiner et al., 2013). Next we validated these profiling data by qRT-PCR of Sirt1 mRNA expression at different ages in RNA isolated from dissected striatum of control and TIF-IA^D1RCre^ mutant mice (Figure 1C). We then compared the expression of SIRT1 protein in the conditional TIF-IA mutant by immunofluorescence and confocal analysis on striatal sections already at 6 weeks (Figure 1D,E). NPM1 specific antibody is used to monitor disruption of nucleolar integrity. In controls the nucleolar protein NPM1 is visible as punctuate staining while in the mutant the signal is diffused in the nucleoplasm (Figure 1D). SIRT1 immunoreactivity is visible in the nuclei in the controls, however, based on semi-quantitative analysis of nuclear signal mean intensity, we find that SIRT1 signal is significantly reduced in the mutant mice (Figure 1D,E). Notably, SIRT1 immunoreactivity is visible in nuclei with intact NPM1 (Figure 1D, arrowhead). These results indicate that inhibition of rDNA transcription and disruption of nucleolar integrity are accompanied by a decreased SIRT1 protein expression, suggesting that there is a crosstalk between SIRT1 protein and nucleolar function.

## Model for the Integration of Sirt1 and Nucleolar Activity

**FIGURE 2:**
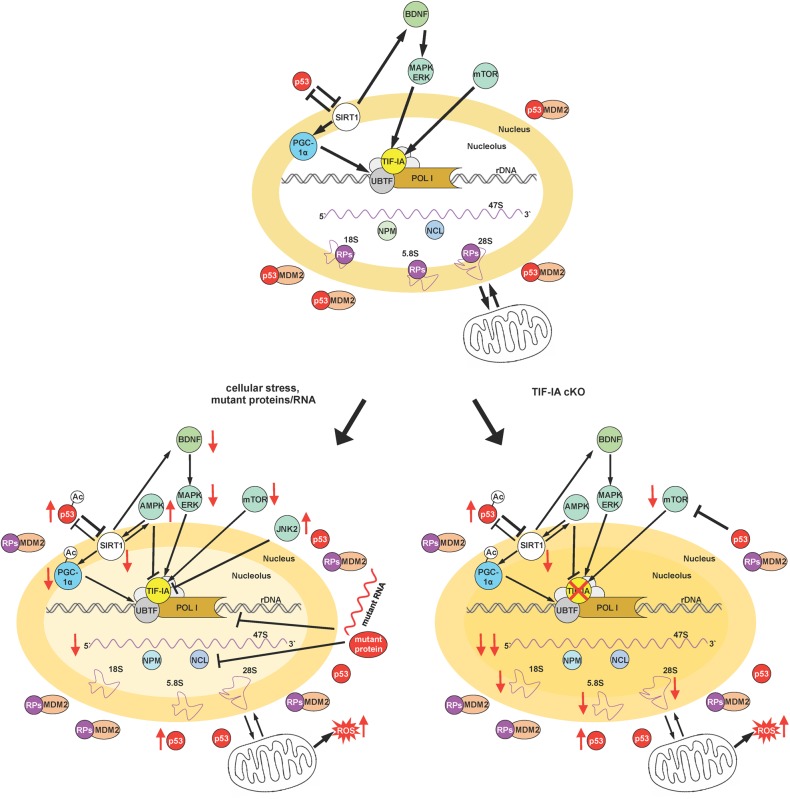
Integration of SIRT1 and nucleolar-mediated signaling. The figure schematizes the proposed model to describe the convergence of various signaling pathways on SIRT1 and TIF-IA to control cellular adaptation to cellular stress signals and their integration in a neuronal context. Depicted are the relationships between neurotrophic signaling, SIRT1 and TIF-IA, as well as rDNA transcription. Moreover the impact of both SIRT1, rRNA synthesis, and p53 activity/stability is also summarized under basal conditions, under cellular stress and in the presence of mutant RNAs and proteins (typical of neurodegenerative diseases), and upon induction of nucleolar stress by the conditional ablation of TIF-IA gene (please see text for a detailed description). Nucleolar stress is visualized by the loss of nucleolar integrity. Arrows indicate activation or induction, while blunt lines denote inhibition, and red arrows decreased/increased level/activity. A thinner line describes a decreased activity of the indicated process.

Based on these and previous findings we propose a model in which SIRT1- and nucleolar-dependent signaling pathways are integrated for the regulation of p53 acetylation and activity upon induction of nucleolar stress (Figure 2). Under basal conditions permissive signals stimulate neuronal growth and function, p53 levels are regulated by proteostatic mechanisms and both SIRT1 and TIF-IA are neuroprotective by regulating rDNA transcription and mitochondrial function (Figure 2, upper panel). Under cellular stress, but also due to accumulation of mutant RNAs and proteins, as in neurodegenerative diseases, rDNA transcription is downregulated as well as its processing, and ribosome biogenesis. Reduced SIRT1 expression results in increased acetylation of p53 and PGC-1α leading to a decreased rDNA transcription with neurotoxic effects. Of note, decreased SIRT1 might also lead to a decreased BDNF-mediated neuroprotection (Figure 2, left panel).

In the absence of TIF-IA, the signaling pathways stimulating rRNA synthesis are strongly impaired, including BDNF signaling. In this model the nucleolar integrity is dramatically affected and nucleolar proteins are released in the nucleoplasm. Here we provide initial evidence of decreased expression of SIRT1 protein at a stage before p53 acetylation that has a pro-apoptotic function, suggesting that this SIRT1 imbalance might play a role in the switch to neuronal death (Figure 2, right panel).

Interestingly both SIRT1 and TIF-IA function maintain p53 activity/level low. Moreover SIRT1 regulates RNA Pol I function either by PGC-1α or by epigenetic modifications and TIF-IA by promoting RNA Pol I recruitment. Furthermore, BDNF converges on TIF-IA activation and SIRT1 may induce via cAMP-response element-binding protein (CREB) the transcription of BDNF (Gao et al., 2010; Gomes et al., 2011; Shen et al., 2018).

The question remains how SIRT1 protein expression is downregulated in the TIF-IA^D1RCre^ mutants. Interestingly, SIRT1 mRNA levels are also increased and SIRT1 protein levels are reduced in post-mortem HD brains and certain transgenic HD mouse models (Pallas et al., 2008; Hathorn et al., 2011; Baldo et al., 2018). One plausible hypothesis is that SIRT1 protein is degraded via the ubiquitin-proteasome pathway as in PD cellular models (Zhang et al., 2018). Another hypothesis implies post-transcriptional mechanisms that involve microRNAs inhibiting SIRT1 mRNA translation. Notably, p53 induces miR-34a expression that blocks SIRT1 and induces apoptosis, cell growth, and senescence (Chua and Tang, 2018). A recent study shows that the connection between p53/miR-34a and SIRT1 is however, altered in some HD mice in association with increased SIRT1 protein (Reynolds et al., 2018). This suggests altered model- and cell-specific regulatory mechanisms and encourages further detailed characterization, including the treatment with proteasome and protein synthesis inhibitors.

## Concluding Remarks and Open Questions

Given the central role of a dysfunctional nucleolus in neuronal homeostasis and its emerging dysregulation in neurodegenerative disorders, therapeutic approaches aiming at promoting SIRT1 activity will need to be tested for their impact on the function and integrity of the nucleolus and the restoration of nucleolar-dependent signaling.

Sirtuin 1 activity is controlled by severe DNA damage as well (Conrad et al., 2016). To dissect the DNA-damage independent effect of nucleolar stress on SIRT1 expression, it would be important to investigate the impact of novel compounds inhibiting RNA Pol I without activating the cellular DNA damage response, such as BMH-21, in a neuronal context (Peltonen et al., 2014; Wei et al., 2018).

A recent study has shown that SIR2 repression is important for monitoring rDNA copy number and for their recovery to a stable level, meaning increased number of rDNA repeats. rDNA repeats can be considered a source of adaptive response to genomic stresses (Salim and Gerton, 2019). Although further analysis is required to establish whether a similar mechanism exists in other organisms, maintenance of genomic integrity at a highly repetitive rDNA region might be essential for cell survival (Iida and Kobayashi, 2018).

Moreover the implications of other members of the sirtuin family remain unknown. Interestingly, SIRT7 is the only nucleolar member of the sirtuin family of NAD+-dependent protein deacetylases, and it coordinates pre-rRNA synthesis and maturation (Ford et al., 2006). Its deacetylase activity is impaired upon cellular stress when SIRT7 is released from the nucleoli so that rRNA synthesis can be downregulated (Chen et al., 2013, 2016). As for now, we cannot exclude that a similar process takes place also in neurons, although it has been in particular shown in dividing cells thus far (Blank and Grummt, 2017).

In perspective, the understanding of the cell-specific link between nucleolar stress and SIRT1 promises a more precise interpretation and prediction of therapeutic benefits.

## Ethics Statement

The procedures involving animal care were approved by the Committee on Animal Care and Use (Regierungspräsidium Karlsruhe) in accordance with the local Animal Welfare Act and the European Communities Council Directives (2010/63/EU and 2012/707/EU).

## Author Contributions

GK, AS, and RP acquired the data. GK, AS, BL, and RP analyzed and interpreted the data. RP contributed to study concept and design and drafted the manuscript. All authors revised the submitted manuscript.

## Conflict of Interest Statement

The authors declare that the research was conducted in the absence of any commercial or financial relationships that could be construed as a potential conflict of interest.

## Abbreviations

AD: Alzheimer’s disease
AMPK: AMP-activated protein kinase
BDNF: brain-derived neurotrophic factor
D1R: dopamine 1 receptor
HD: Huntington’s disease
JNK2: c-jun N-terminal kinase 2
mTOR: mechanistic target of rapamycin
NAD+: nicotinamide adenine dinucleotide
NCL: nucleolin
NPM1: nucleophosmin 1
PD: Parkinson’s disease
PGC-1α: peroxisome proliferator-activated receptor-gamma co-activator-1 alpha
qRT-PCR: quantitative real-time PCR
rDNA: ribosomal DNA
RNA Pol I: RNA polymerase I
rRNA: ribosomal RNA
SIRT1: sirtuin 1
TIF-IA: transcription initiation factor-IA
UBTF1: upstream binding transcription factor 1
